# Differential Expression of IFN-*γ*, IL-10, TLR1, and TLR2 and Their Potential Effects on Downgrading Leprosy Reaction and Erythema Nodosum Leprosum

**DOI:** 10.1155/2019/3405103

**Published:** 2019-11-07

**Authors:** Douglas Eulálio Antunes, Isabela Maria Bernardes Goulart, Mayara Ingrid Sousa Lima, Patrícia Terra Alves, Paula Cristina Brígido Tavares, Luiz Ricardo Goulart

**Affiliations:** ^1^National Reference Center for Sanitary Dermatology and Leprosy, Clinics' Hospital, Federal University of Uberlandia, Uberlandia, MG 38401-404, Brazil; ^2^Department of Biology, Federal University of Maranhão, São Luis, MA 65080-805, Brazil; ^3^Institute of Biochemistry and Genetics, Federal University of Uberlandia, Uberlandia, MG 38400-902, Brazil; ^4^Department of Medical Microbiology and Immunology, University of California, Davis, Davis, CA 95616, USA

## Abstract

Leprosy reactions are acute immunological events that occur during the evolution of chronic infectious disease causing neural damage and disabilities. A study using blood samples of 17 leprosy reaction patients and 17 reaction-free was carried out by means of associations between antigens, receptors, and expression of cytokines, using path analysis providing new insights into the immunological mechanisms involved in triggering leprosy reactions. Toll-like receptors (TLR) such as TLR1 and TLR2, presented balanced expression in the reaction-free multibacillary (MB) group (TLR1: 1.01 ± 0.23, TLR2: 1.22 ± 0.18; *p* = 0.267). On the other hand, downgrading type 1 reaction (T1R) (TLR1: 1.24 ± 0.17, TLR2: 2.88 ± 0.37; *p* = 0.002) and erythema nodosum leprosum (ENL) (TLR1: 1.93 ± 0.17, TLR2: 2.81 ± 0.15; *p* = 0.004) revealed an unbalance in relation to the expression of these receptors. When the path analysis was approached, it was noted that interleukin 10 (IL-10) expression showed a dependence relation with phenolic glycolipid I (PGL-I) in downgrading T1R (direct effect = 0.503 > residual effect = 0.364), whereas in ENL, such relationship occurred with lipoarabinomannan (LAM) (direct effect = 0.778 > residual effect = 0.280). On the contrary, in the reaction-free leprosy group, interferon-gamma (IFN-*γ*) levels were dependent on the association between TLR2 and TLR1 (0.8735). The high TLR2 expression associated with IL-10 levels, in the leprosy reaction groups, may be hypothetically related to the formation of TLR2/2 homodimers and/or TLR2/6 heterodimers linked to evasion mechanisms in downgrading reactions and pathophysiology of ENL.

## 1. Introduction

Leprosy reactions are acute immunological events that overlap the chronic infection caused by *Mycobacterium leprae* (*M. leprae*). The antigenic components of this bacillus are the potential triggers of these reactions that affect in different degrees the peripheral nerves causing physical disabilities [1]. These immunological events are classified into type 1 (T1R) and erythema nodosum leprosum (ENL), affecting different clinical forms of the disease before, during, and after treatment [2].

The type 1 reaction (T1R), subdivided in upgrading and downgrading, is a delayed hypersensitivity reaction against components of *M. leprae*, whose the affected clinical forms are borderline tuberculoid (BT), borderline borderline (BB), and borderline lepromatous (BL) [3].

The upgrading and downgrading reactions are clinically indistinguishable, characterized by the presence of oedema and erythema in preexisting skin lesions, appearance of new skin lesions with classic inflammatory signs, and neuritis associated with sensory and motor alterations [4]. On the other hand, such reactions may be differentiated by histopathology, the profile of the immunological response, and temporality of the occurrence of these events [4].

The upgrading reaction, also called reverse reaction, occurs after administration of multidrug therapy (MDT), in which the type 1 helper (Th1) cytokine pattern (interleukin-1*β* [IL-1*β*], tumor necrosis factor-alpha [TNF-*α]* IL-2, and interferon-gamma [IFN-*γ*]) is found in patient lesions, in addition to elevation of TNF-*α*, IFN-*γ*, and IL-17F in the serum of these patients and other markers such as interferon gamma-induced protein 10 (IP-10), vascular endothelial growth factor (VEGF), and chemokine 10 (CXCL10) [5–8]. The T1R guarantees resistance against *M. leprae*, leading to migration in the clinical spectrum of the disease of those borderline individuals to the tuberculoid pole, reducing, finally, the bacilloscopic and morphological indices [9].

On the contrary, the downgrading reaction occurs before MDT and after treatment in relapse cases, representing an immunological activity directed against nonessential antigenic determinants of *M. leprae* survival. Thus, it may be observed in downgrading reaction the increase in the number of bacilli, B lymphocyte levels, and immunoglobulin gamma (IgG) antibodies, besides the low levels of natural killers and T cells [4, 10, 11]. Furthermore, the immunological profile of this reaction allows evasion mechanisms of the bacillus favoring the migration of borderline individuals towards the lepromatous leprosy (LL) pole in the clinical spectrum of the disease [9, 12].

Regarding the type 2 reaction, also called erythema nodosum leprosum (ENL), it represents a type III hypersensitivity reaction caused by the deposition of immune complexes in the joints, skin, endothelium, and other body structures, affecting 10% of BL and 50% of LL [13]. The main clinical presentation of this reaction are erythematous nodules in the skin, in addition to systemic symptoms such as fever, malaise, arthralgia, myositis, iridocyclitis, orchitis, glomerulonephritis, and laboratory abnormalities, such as neutrophilia and high C-reactive protein [13–15]. The proinflammatory component was associated with the immunopathology of ENL in several studies, since patients presented an increasing of CD4^+^ T lymphocytes and a reduction in the levels of CD8^+^ T cells in the blood when compared with reaction-free LL controls [16]. Elevated levels of circulating TNF-*α* as well as expression of IL-6 and IFN-*γ* were present in the serum and cutaneous lesions of ENL patients [17–19].

The *M. leprae* presents in its cell wall a glycolipid called lipoarabinomannan (LAM) and in its capsule the phenolic glycolipid I (PGL-I), two important surface molecules that are recognized mainly by Toll-like receptor 1 (TLR1) and 2 (TLR2) that associate to form the heterodimer TLR1/2. This heterodimer activates pathways that control dissemination of intracellular microorganisms influencing, therefore, components of innate and adaptive immunities [20].

A probable TLR2/2 homodimer was hypothesized and associated with IL-10 synthesis in mycobacteria. Although IL-10 plays an important role in the control of the inflammatory process, its elevation inhibits the synthesis of proinflammatory cytokines, which facilitates the survival and persistence of pathogens such as *M. leprae*, functioning, thereby, as an evasion mechanism [21].

Therefore, by means of gene expressions, serological data, and a causal model, this study has aimed hypothesizing the presence of an unbalance between the TLR1 and TLR2 expressions associated to high bacillary loading and IL-10 expression in leprosy reactions, which, consequently, are favorable to survival of bacillus and the occurrence of these events.

## 2. Material and Methods

### 2.1. Type of the Study and the Sample

This is a cross-sectional study, in which the sample was composed of 34 leprosy patients, being 17 with leprosy reactions (7 T1R and 10 ENL) and 17 reaction-free leprosy patients (8 paucibacillaries and 9 multibacillaries). All patients selected to this research were diagnosed by experts on leprosy according to the clinical, histological, and immunological criteria of Ridley and Jopling [2].

### 2.2. Place of the Study

The sample was selected according to inclusion and exclusion criteria for a long and sufficient period. The data collection was performed at the National Reference Center for Sanitary Dermatology and Leprosy (CREDESH) of the Federal University of Uberlândia (UFU), MG, Brazil, from 2014 to 2016.

### 2.3. Inclusion and Exclusion Criteria

The inclusion criteria were participants older than 18 years, leprosy patients affected by leprosy reactions before, during, or after treatment, and leprosy patients not affected by leprosy reactions (composing the reaction-free leprosy group).

Exclusion criteria were individuals with comorbidities as other chronic or acute diseases, the use of thalidomide and/or steroid therapy, and the use of immunotherapies and analogues.

### 2.4. Data Collection

In the reaction group, biological samples from each patient were collected once on the first day of clinical exacerbation of the leprosy reaction. Therefore, samples were obtained before, during, and/or after MDT.

Regarding the reaction-free group, all patients had biological samples collected before starting MDT.

### 2.5. Clinical and Epidemiological Variables

Variables such as clinical form; operational classification; sex, age group; bacilloscopic index; disability grade (DG); number, distribution and characteristics of cutaneous lesions and affected nerves in the diagnosis were obtained. The clinical evaluation of leprologists allowed quantifying the number of cutaneous lesions and affected nerves (evidenced by physical examination and electroneuromyography) in the diagnosis, besides classifying the disability grade of individuals from 0 to 2 [22].

### 2.6. ELISA anti-PGL-I Serology

The presence of anti-PGL-I and LAM antibodies reflects bacillary load and helps classifying clinical forms. The detection of IgM antibodies in anti-PGL-I serological tests, instead of IgG, increases sensitivity and influences performance in the serological test among patients with PB and MB leprosy, besides IgM to be produced in acute phase of infection [23].

The native PGL-I isolated by organic extraction of *M. leprae*-infected armadillo tissues from which the bacteria had been purified and utilized in PGL-I ELISA was obtained from Colorado State University through the NIH/NIAID Leprosy Contract N 01 AI 25469.

For the PGL-I antibody detection ELISA assays, microtiter plates (MaxiSorp-NUNC®) were covered with native PGL-I diluted in phosphate-buffered saline (PBS), at concentration of 0.2 *μ*g/ml. Serum samples were added in duplicate using a dilution of 1 : 100 (native PGL-I) in PBS/BSA 1%, incubated for 1 hour at 37.8°C, and subsequently washed. The anti-human IgM-peroxidase conjugate (Sigma Chemical Co., St. Louis, MO) was added to the plates in the dilution of 1 : 10.000 (PGL-I ELISA) and 1 : 2.000 (ND-O-HSA). The substrate o-phenylenediamine dihydrochloride (OPD, Sigma) enzyme substrate was added to the plates and incubated at room temperature for 10 minutes in the dark chamber. The reaction was stopped by the addition of H_2_SO_4_ 4 N. The optical density (OD) was obtained in a microplate reader (Thermo Plate, TP-Reader, Rayto Life and Analytical Sciences Co. Ltd, Germany) at 492 nm. Two positive and three negative controls were included in each plate.

### 2.7. ELISA anti-LAM Serology

Regarding anti-LAM, studies have revealed that IgG is the predominant circulating antibodies against *M. leprae* antigens [24, 25].

About the method, the LAM antibody, a monoclonal antibody derived from the cell wall of *M. leprae* extracted from a pool of an infected armadillo liver and spleen tissue, was sensitized 96-well high affinity plates (MaxiSorp, Nunc®) with 50 *μ*l Native LAM (BEI Resources, NR-19348) diluted in carbonate/bicarbonate buffer (50 *μ*l Native LAM 100 *μ*g/ml diluted in 4950 *μ*l of carbonate/bicarbonate buffer, pH 9.6); the plates were incubated overnight in a cold room at 4°C; four washes were performed with 0.05% PBST (200 *μ*l/well), and serum samples diluted in 5% PBS/BSA (1 : 5) were added in triplicate. The plates were incubated for 1 h at 37°C and, after five washes with 0.05% PBST, were added 50 *μ*l of peroxidase-labeled anti-IgG diluted 1: 1000 in PBS/BSA and incubated for 1 h at 37°C; after six washes with 0.05% PBST, the plates were exposed 50 *μ*l of OPD solution for 5 min (2 mg OPD+5000 *μ*l buffer citrate+2 *μ*l H_2_O_2_), and the reaction was then quenched with 20 *μ*l/well of sulfuric acid (H_2_SO_4_ 2N). The plates were read on a microplate reader (TP-Reader, Thermo Plate®) at a wavelength of 492 nm.

Antibody titers were expressed as direct values of optical density and subsequently subjected to statistical normalization for a percentage scale that maintained the ratio between differences in antigen expression levels.

### 2.8. RNA Isolation, cDNA Synthesis, and Real Time qPCR

RNA from blood was extracted using the TRIzol® LS reagent (Life Technologies, Carlsbad, CA, USA) according to the manufacturer's instructions. The concentration and quality of the RNA were determined by ultraviolet absorbance and electrophoresis. Complementary DNA (cDNA) was generated by reverse transcription (MMLV, Life Technologies, Carlsbad, CA, USA) using 1 *μ*g of RNA, according to the manufacturer's instructions.

Real-time quantitative polymerase chain reaction (real-time qPCR) with the thermocycler ABI PRISM 7300 (Applied Biosystems, USA) was used for gene expression analyses by using the TaqMan Universal PCR Master Mix to quantify the TLR1, TLR2, IFN-*γ*, and IL-10 genes in the peripheral blood of patients. The qPCR reaction was developed with a final volume of 12 *μ*l, with the following reaction mix: 6 *μ*l of Master Mix, 0.2 *μ*l of specific set of TaqMan primers and probe, 5 *μ*l of the cDNA, and 0.8 of distilled water-free RNase. Amplification conditions were those recommended by the manufacturer. All reactions were performed in triplicate, and probes used were TLR1 (Hs00413978_m1), TLR2 (Hs01014511_m1), IL-10 (Hs00961619_m1), IFN-*γ* (Hs00989291_m1), and glyceraldehyde 3-phosphate dehydrogenase (GAPDH; Hs03929097_q1) which was used as the endogenous control. Cycling conditions were 50°C for 2 min and 95°C for 10 min, followed by 40 cycles of 95°C for 15 s and 60°C for 1 min. For mRNA quantification, we have used the method 2^-*ΔΔ*Ct^ as described elsewhere [26] and the expression data are represented in fold change.

### 2.9. Sample Size Calculation

We calculate the sample size of this study using the software G^∗^ Power (version 3.1.9.2, for windows) to reduce the costs and prove the statistical hypothesis. In order to obtain the effect size, it used the correlation coefficient (0.47) obtained from the relation between the bacilloscopic index and the anti-PGL-I IgM ELISA index of a previous pilot study. The statistical significance level alpha was 5% (0.05), and the power of the test was 0.85 (85%). Our sample size, considering the above parameters, was 34 individuals.

### 2.10. Statistical Analyses

The Shapiro-Wilk test was used to test the normality of data distribution. In comparing the two groups, reactional and reaction-free, the Student's *t*-test was performed to detect differences between means of serological markers and immunological variables. Regarding these previously quoted variables, the Analysis of Variance (ANOVA test) was chosen to analyze the differences between more than three groups. The binomial test was employed to prove the control of confounding factors related to epidemiological variables, by means of a comparison among the proportion of reactional and reaction-free cases. To verify the magnitude of the association among variables in the sample, the Pearson's correlation matrix was calculated.

### 2.11. Path Analysis

The path analysis, based on multiple linear regression, is the most robust test used in multivariate statistics [27]. Thus, direct and indirect effects were quantified between the dependent and independent variables. Interpretation of the path model was done as follows: direct effect is represented by unidirectional arrows (←), with their respective values (estimates), starting in the independent variable towards the dependent variable; bidirectional arrows (↔) and their respective values represent the correlation between two independent variables; the indirect effect of two independent variables on the dependent is represented by the combination of both bidirectional and unidirectional arrows (↔ . ←), whose exact values can be calculated by multiplication of these two numerical estimates [28].

According to Singh and Chaudhary criteria [29], the independent variable (*x*) influences the dependent variable (*y*) indirectly only, if the direct effect of variable (*x*) on (*y*) was less than the residual effect (*pε*) which is less than the total effect (*r*_*yx*_), summed up by ∣*p*_*yx*_∣ < *pε* < *r*_*yx*_, or the independent variable (*x*) can influence the dependent (*y*) inferring direct causal relation, if the direct effect of the variable *x* on *y* is bigger than the residual effect, finally represented as ∣*p*_*yx*_∣ > *pε*.

When one or more variables are considered independent and dependent variables, concomitantly, it means that there is more than one causal model, this way we have a path analysis in the chain [30].

It was used for statistical calculations the GraphPad Prism 7.0 (GraphPad Software, San Diego, CA, USA) and the Software for Experimental Statistics in Genetics® (GENES Software, Lavras, MG, Brazil) specifically designed for path analysis [31]. The significance level *α* was 5% for all analyses.

### 2.12. Ethical Considerations

This study was carried out in accordance with the recommendations of “Guidelines of the National Board on Research Ethics (CONEP)” with written informed consent from all subjects. All subjects gave written informed consent in accordance with the Declaration of Helsinki. The protocol was approved by UFU Research Ethics Committee under the number 633.052/2014.

## 3. Results

### 3.1. Clinical and Epidemiological Characterization

The study was formed by 34 patients with leprosy, divided into two groups, reactional and reaction-free patients, each one with 17 individuals. Among all samples, 76.4% (26/34) were classified as multibacillary (MB) and 23.6% (8/34) as paucibacillary (PB), in which 100% of leprosy reaction cases were MB (17/17) and 47% (8/17), in the reaction-free group, were PB (Table 1).

In the leprosy reaction group, the most frequent clinical forms were the LL 53% (9/17) followed by BB with 23.5% (4/17) according to Table 1. Still, analyzing this group, 41.2% (7/17) showed T1R, while 58.8% (10/17) had ENL (Table 1). It was noted that 29.4% (5/17) of the reactions occurred before treatment, all of T1R, and 70.6% (12/17) after treatment (Table 1). Regarding the disability grade, the zero degree predominated in 61.7% (21/34) of the individuals in the sample, whose highest frequency was present in the reaction-free group representing 38.2% (13/34) of the sample (Table 1). There was a predominance of males in both groups (58.8%, 10/17) (Table 1).

The mean age of the leprosy reaction group was 45 years, while the reaction-free group was 47 years. In the sample, ages from 35 to 44 years and from 45 to 54 years were predominant, with frequencies of 29.4% and 23.6%, respectively. However, in comparison between leprosy reaction and reaction-free groups, concerning the age group, the binomial test showed that there was no difference between age group proportions, which represents, therefore, the control of confounding factors associated with this variable (25-34, *p* = 0.527; 35-44, *p* = 1.00; 45-54, *p* = 1.00; 55-64, *p* = 0.302; and ≥65, *p* = 0.392).

### 3.2. Laboratorial Analyses

**Figure 1:**
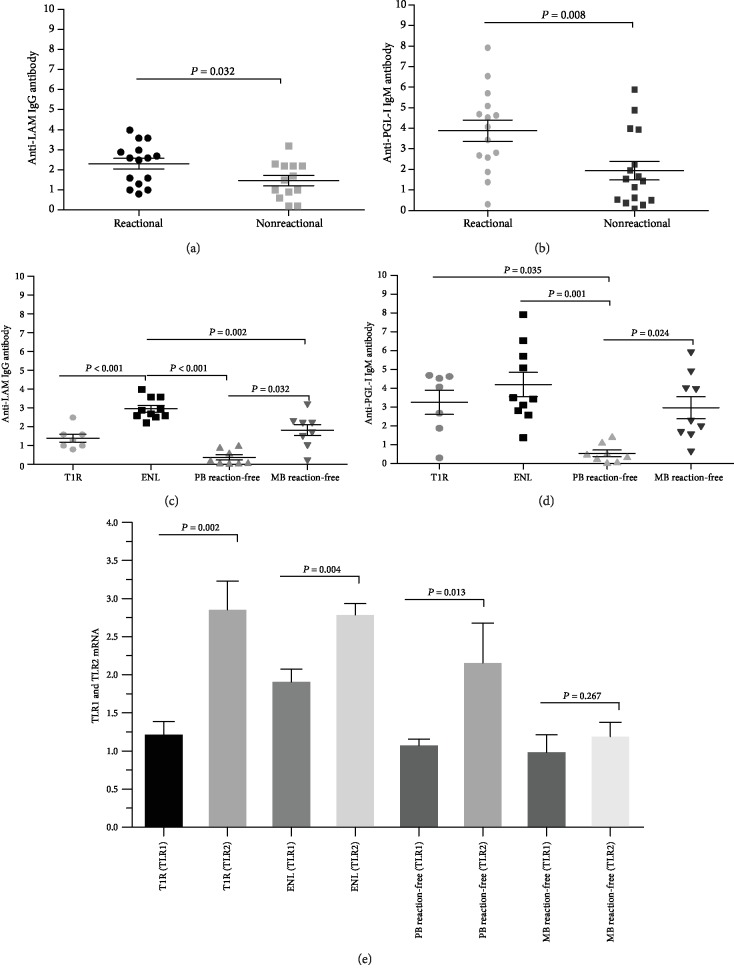
Serological markers (LAM and PGL-I) and TLR 1 and 2 in the peripheral blood of leprosy reaction and reaction-free leprosy patients. (a) Comparison between mean levels of anti-LAM IgG by ELISA (Enzyme-Linked Immunosorbent Assay) in the leprosy reaction and reaction-free leprosy patients. (b) Comparison between mean levels of anti-PGL-I IgM by ELISA in the leprosy reaction and reaction-free leprosy patients. (c) Comparison between mean levels of anti-LAM IgG by ELISA in the reactional and reaction-free leprosy patients PB and MB. (d) Comparison between mean levels of anti-PGL-I IgM by ELISA in the reactional and reaction-free leprosy patients PB and MB. (e) Comparison between TLR1 and TLR2 mRNA gene expression in the T1R, ENL, reaction-free leprosy patients PB and MB. The RNA expression was represented in fold change in relation to the endogenous control.

It was verified that the mean (*m*) and the standard error of the mean (SEM) of anti-LAM in the leprosy reaction group (2.30 ± 0.26) was significantly higher than reaction-free group (1.46 ± 0.25) (*p* = 0.032) (Figure 1(a)). The anti-PGL-I presented significantly higher levels in the leprosy reaction group (3.88 ± 0.52) than reaction-free (1.93 ± 0.44) (*p* = 0.008) (Figure 1(b)).

The Figure 1(c) shows the results after stratifications of the groups. The groups with ENL (3.10 ± 0.18) and reaction-free leprosy MB (1.82 ± 0.28) presented higher levels of anti-LAM IgG; in addition, the levels of this antibody in ENL group differed from all others as observed in Figure 1(c).

As for the levels of anti-PGL-I IgM, the comparative analysis between reaction-free leprosy PB (0.61 ± 0.18) and the groups T1R (3.25 ± 0.63), ENL (4.43 ± 0.79) and reaction-free leprosy MB (2.96 ± 0.58) showed a significant difference among levels expression of that antibody as shown in Figure 1(d).

The TLR1 and TLR2 expression levels were compared in all groups showing that these receptors in reaction-free leprosy MB group there were balanced expression (TLR1: 1.01 ± 0.23, TLR2: 1.22 ± 0.18; *p* = 0.267) (Figure 1(e)).

However, the groups with T1R (TLR1: 1.24 ± 0.17, TLR2: 2.88 ± 0.37; *p* = 0.002) and ENL presented unbalance among the expressions of these receptors (TLR1: 1.93 ± 0.17, TLR2: 2.81 ± 0.15; *p* = 0.004) (Figure 1(e)).

**Figure 2:**
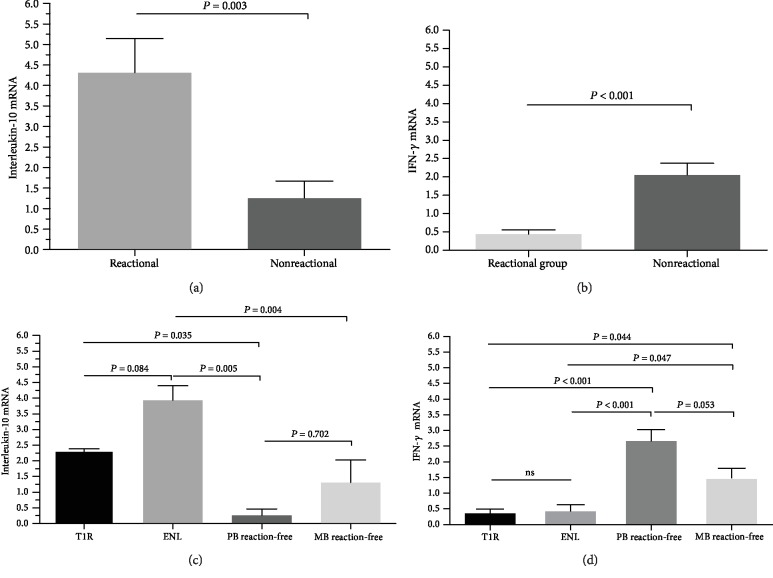
IL-10 and IFN-*γ* expression in leprosy reaction and reaction-free leprosy patients. (a) Comparison between IL-10 expression in the reactional and reaction-free leprosy. (b) Comparison between IFN-*γ* expression in the reactional and reaction-free leprosy patients. (c) Comparison between IL-10 expression in the reactional and reaction-free leprosy patients PB and MB. (d) Comparison between IFN-*γ* expression in the reactional and reaction-free leprosy patients PB and MB.

For the cytokine analyses, the IL-10 expression was relatively higher in the leprosy reaction group (4.31 ± 0.83) when confronted with reaction-free leprosy patients (1.25 ± 0.53) (*p* = 0.003) (Figure 2(a)). In relation to the expression of IFN-*γ* in the peripheral blood, higher levels were observed in the reaction-free leprosy patients (2.05 ± 0.32) when compared to the leprosy reaction group (0.43 ± 0.12) (*p* < 0.001) (Figure 2(b)). To better understand the expression levels of IL-10, all groups were subdivided, as previously mentioned. It was observed, according to Figure 2(c), that both the group with T1R (2.31 ± 0.09) and ENL (3.93 ± 0.46) expressed relatively higher levels of IL-10 with significant differences when compared to reaction-free leprosy PB (0.25 ± 0.21) and MB (1.30 ± 0.72).

IFN-*γ* was also analyzed in the 4 groups (Figure 2(d)), with high expression in the reaction-free leprosy PB (2.70 ± 0.37) and MB (1.49 ± 0.30) in contrast to the low expression in the groups with T1R (0.39 ± 0.14) and ENL (0.46 ± 0.20), with significant differences among the reaction-free leprosy PB group and the groups with T1R (*p* < 0.001) and ENL (*p* < 0.001).

### 3.3. Path Analysis

The *Pearson's* correlation matrix in the leprosy reaction group demonstrated positive correlations among the dependent variable IL-10 and the independent variables TLR2 (*r* = 0.89; *p* < 0.001), anti-LAM (*r* = 0.55; *p* = 0.043), anti-PGL-I (*r* = 0.70; *p* = 0.004), number of injured nerves (*r* = 0.62; *p* = 0.018), and number of skin lesions (*r* = 0.69; *p* = 0.005) (Table 2).

Positive and significant correlations between IFN-*γ* expression and both TLR1 (*r* = 0.74; *p* = 0.023) and TLR2 (*r* = 0.78; *p* = 0.013) in the reaction-free leprosy group were shown in Table 3.

**Figure 3:**
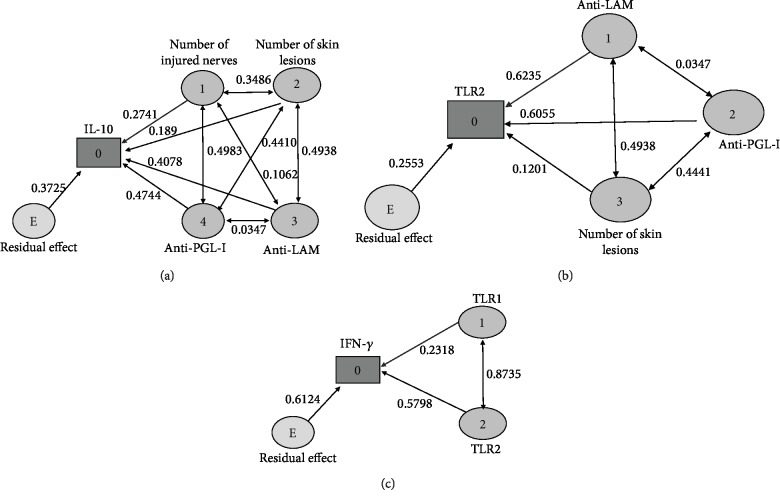
Path diagrams of the leprosy reaction and reaction-free leprosy groups. (a) A path diagram indicating the relationships among independent variables involved in diagnosis, number of skin lesions at diagnosis, anti-LAM, and anti-PGL-I influencing the variation of the dependent variable IL-10 in the reaction group. (b) A path diagram showing the relationships among independent variables, number of skin lesions at diagnosis, anti-LAM, and anti-PGL-I promoting the variation in TLR2 expression in the reaction group. (c) A path diagram involving independent variables TLR1 and TLR2, which induced variation in the dependent variable IFN-*γ* in the reaction-free leprosy group. The path analysis demonstrated dependence relation among dependent variable (0) and independent variables with direct effect greater than the residual effect.

Figure 3(a) demonstrated the direct effects of anti-LAM (0.407) and anti-PGL-I (0.474) on IL-10 (dependent variable), which were greater than the residual effect (0.372), demonstrating the causal relationship among these variables and IL-10 expression in the leprosy reaction groups.

Figure 3(b) shows the direct effects of anti-LAM (0.623) and anti-PGL-I (0.605) on TLR2 (second dependent variable in the reactional group), which were greater than the residual effect of this model (0.255).

On the other hand, Figure 3(c) demonstrates that the IFN-*γ* expression can be influenced by association between TLR1 and TLR2 (0.873) that was greater than the residual effect (0.612) of this model.

**Figure 4:**
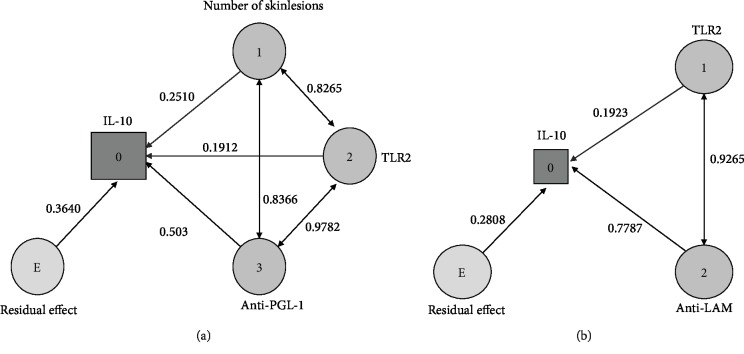
Path diagrams of type 1 leprosy reactions and ENL. (a) A path diagram indicating the dependence relationship between IL-10 (dependent variable) and anti-PGL-I in the group with T1R after extratification of the groups. (b) Path analysis pointing to the dependence relationship between IL-10 (dependent variable) and anti-LAM levels in ENL after extratification of groups.

Figure 4(a) shows the causal diagram in T1R, whose direct effect of anti-PGL-I (0.503) and indirect effect of TLR2 via anti-PGL-I (0.488) on IL-10 were greater than the residual effect (0.364).  Figure 4(b) shows the causal model in ENL, whose direct effect of anti-LAM (0.778) and indirect effect of TLR2 via anti-LAM (0.721) on IL-10 expression were greater than the residual effect of this model (0.280).

## 4. Discussion

The present study demonstrates, through path analysis, the dependence relationship between the major antigens of *M. leprae* and receptors of innate immunity (especially TLR2) indicating a possible key role in triggering the reactional states, in fact contributing with an immunosuppressive immune response favorable to survival and bacillary multiplication.

The association between the leprosy reaction group and the operational classification in the present study showed that MB patients presented the highest potential to develop leprosy reactions, independently of the type of reaction, which is corroborated by other research [32]. In this study, the ENL was the most frequent in the leprosy reaction group, probably because most MB patients are from the borderline leprosy (BL) and LL clinical forms. This study differs from the prevalence demonstrated in other studies, in which the borderline tuberculoid (BT) and T1R were the most prevalent [33]. It is noteworthy that most of the T1R occurred before treatment, which confirms a downgrading T1R and the presence of an immunosuppressive profile associated to an increase in IL-10. With respect to ENL, this reactions occurred after treatment related to the presence of IL-10 playing an important role in the immunopathogenesis of this event [4, 11, 34]. Regarding the disability grade in diagnosis, those individuals not affected by leprosy reactions will mostly have a degree of disability of zero due to the relationship between leprosy reactions and neural damage [35]. The prevalence of males in the groups is in agreement with another research that associates the concern of women with health status to the early diagnosis of the disease [36].

The mean age in the leprosy reaction group in this study is also concordant with previous reports [37, 38].

Higher levels of anti-LAM in the leprosy reaction group is due to the presence of great number of individuals with ENL, since LAM is involved in the formation of immune complexes and in the pathogenesis of erythema nodosum leprosum [39]. High levels of anti-PGL-I in leprosy reactions, independently on the type of reaction, ratify that this antigen is a risk marker for the occurrence of reactions, during treatment and after discharge from MDT, corroborating with previous studies that showed positive serology anti-PGL-I as a risk factor to the reactional condition [3, 7, 40].

When analyzing and comparing the expression of TLR1 and TLR2 in the same group, our findings demonstrated differences in these expressions, mainly in the reactional groups, in contrast to the quantitative balance of these receptors in the reaction-free leprosy MB group. These differences can indicate that there may be a signaling pathway-dependent heterodimer TLR1/2 in these patients determining the immune response to the pathogen [20].

As an additional evidence, we have also shown that TLR1 and TLR2 expression levels presented no differences in the reaction-free leprosy MB group, a high-risk group to develop leprosy reaction; however, they were not affected by this reactions during the research.

Studies have quoted the importance of the physical interaction between TLR1 and TLR2 in the recognition of mycobacterial antigens and consequent activation of nuclear factor kappa B (NF-*κ*B) inducing the synthesis of proinflammatory cytokines [20, 41]. On the contrary, using knockout macrophages (TLR1^−/−^ or TLR2^−/−^), authors demonstrated that in the absence of one of these receptors there was damage in the activation of the NF-*κ*B and, thus, low levels of TNF-*α* [42]. This association between TL1/2 corroborates with our results, mainly in the reaction-free groups, which suggests heterodimer formation and activation that leads to IFN-*γ* expression, a specific response to mycobacterial antigens [43, 44].

The differential expression among TLRs in the leprosy reaction groups, T1R and ENL, hypothetically suggests that the TLR2/2 homodimer formation may mediate the production of IL-10 [21]. Authors have hypothesised that prolonged TLR2/2 homodimer signaling, induced by mycobacterial components, limits the activation of mitogen-activating protein kinase (MAPK) pathways by inhibiting phagolysosome fusion and antigen presentation by MHC class II. In addition, this mechanism promotes the synthesis of anti-inflammatory cytokines, such as IL-10 and transforming growth factor *β* (TGF-*β*), which in turn, block the activation of NF-*κ*B [21].

Although the hypothetical TLR2/2 homodimer mechanism has been proposed for *M. tuberculosis*, it still requires a functional validation. However, current molecular techniques do not allow to prove the existence of homodimers involving Toll-like receptors [21].

**Figure 5:**
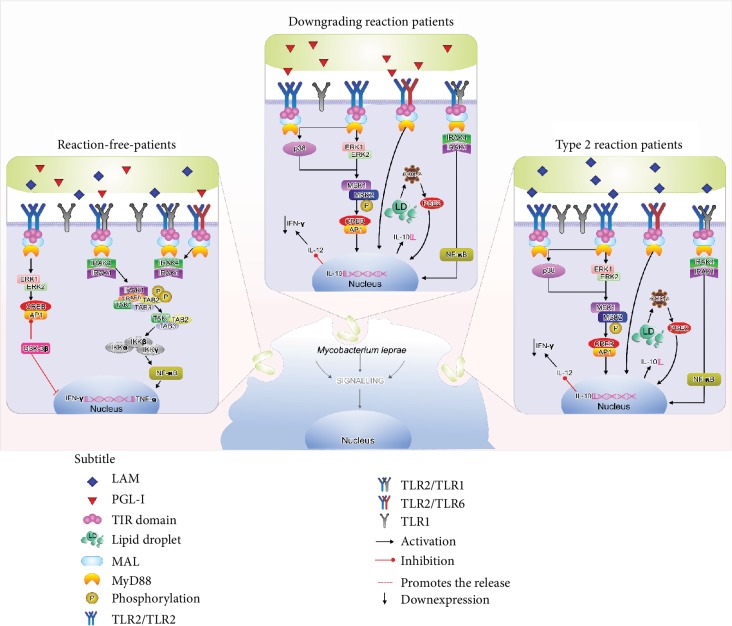
*M. leprae* and TLR interactions. Hypothetical mechanism of leprosy reactions according to evidences of associations between TLR2 and TLR1 receptors, the recognition of LAM and PGL-I markers, and the induction of IL-10 and IFN-*γ* expression. Reaction-free leprosy patients, mainly MB, present balanced production of TLRs (TLR1/2) that induces IFN-*γ* production through NF-*κ*B activation, consequently with a better control of bacillary dissemination. On the other hand, patients with downgrading and ENL present an increased levels of TLR2, which may favor TLR2/2 homodimer formation and association of TLR2/6, which leads to IL-10 production, inhibiting the proinflammatory response and consequently causing bacillary spreading and higher risk of leprosy reactions.

These events are represented in a hypothetical immunological pathway in Figure 5.

Interestingly, using cause and effect diagrams in the leprosy reaction group, we have shown that there may be a hypothetical immunological pathway involving TLR2, LAM, and PGL-I antigens, which was associated with the presence of IL-10, leading to a cellular immune response associated with the lepromatous leprosy pole of the disease [45]. Even though proinflammatory cytokines may contribute to demyelination, in our study, the number of injured nerves was associated with IL-10 expression according to previous studies using a rat Schwann cells (SCs)/axon coculture system and T and B cell-deficient (Rag1^−/−^) mice, which reported rapid demyelination following adherence of *M. leprae* to SCs in the absence of immune cells. Nerve injury may be related to a mechanism dependent on PGL1 as observed in this present study, that is, *M. leprae* is sufficient to induce demyelination [46, 47].

According to previous results and hypothesis, in this present study, higher TLR2 expression in the reactional group, mostly in T1R, may be associated to TLR2/2 homodimer formation and association of TLR2/6 inducing a Th2 profile, while the TRL2/1 heterodimers may be occurring in reaction-free leprosy patients [28, 41, 48, 49]. The unbalanced immune response may explain why some patients with the same clinical form and bacilloscopic indexes will present different clinical outcomes.

To reinforce our hypotheses, studies have also shown that viable *M. leprae* can influence the formation of lipid droplets that lead to prostaglandin E_2_ (PGE_2_) production, which is involved in the synthesis of IL-10 in a TLR2-dependent pathway [48, 50, 51].

We cannot rule out that live bacilli can still induce IL-10 expression by interacting with other receptors, like the leukocyte-Ig-like receptors (LILR) and dendritic cell-specific ICAM-grabbing nonintegrin (DC-SIGN) [52, 53].

Although the predominance of IL-10 rather than IFN-*γ* in these reactions seems incomprehensible, most of the patients with T1R had downgrading reaction, whose bacillary viability may favor cell-mediated immunity, but not as effective as in those individuals affected by upgrading reaction [4, 6, 11, 54]. Regarding ENL, studies have reported the elevation of IL-10 in this reaction that may be explained by its ability to stimulate B cell proliferation and differentiation, which in turn secrete immunoglobulins in its membrane and subsequently, will activate components in the formation of immune complexes. In accordance with the above, high levels of anti-PGL-I IgM, anti-LAM IgG, B lymphocytes in skin lesions, and peripheral blood were pointed out as markers for ENL [55, 56]. In spite of this hypothesis is not proven for leprosy, we cannot fail to highlight another possible role of IL-10 related to its proinflammatory activity, which such cytokine under the action of IFN-*α* will activate the signal transducer and activator of transcription 1 (STAT1) inducing synthesis of chemokine 9 (CXCL9) and chemokine 10 (CXCL10) [57–59].

An important study reported, after cell stimulation with IL-10, the synthesis and elevation of neopterin, whose concentrations increase in the presence of IFN-*γ*, a proinflammatory cytokine [59]. This idea can be reinforced with studies that showed the presence of neopterin as a marker for the occurrence of T1R upgrading and ENL reactions [60].

In T1R, PGL-I was the main agonist that was associated with TLR2 and IL-10 expression. A previous study has narrated that patients with T1R have higher levels of cluster of differentiation 14 (CD14), a macrophage surface marker that concentrates and distributes triacyl-lipopeptides to TLR2 and TLR1, which leads to the hypothesis that this molecular marker, CD14, facilitates the association between PGL-I and TLR2 [61, 62]. The associations among LAM, TLR2, and IL-10 in ENL are in agreement with other studies that have indicated LAM as the main molecule associated to the pathogen that activates the complement and acting in an active way in the formation of these immune complexes [63].

These reactions can be triggered by multiplication of persistent bacilli before and after MDT. On the other hand, during treatment, these events can be related to fragmented mycobacterial products unleashing reactional states [64].

## 5. Conclusions

Finally, we showed an unbalance in the expressions of TLR1 and TLR2, in the leprosy reaction groups, in contrast to reaction-free leprosy MB, the group which presented a balance in these expressions. Thus, we conclude and hypothesized, in reactional groups, a possible signaling pathway favoring the formation of TLR2/2 homodimers, association of TLR2/6, and consequently, greater expression of IL-10, which may favor bacillary survival and the occurrence of these events. The understanding of this unbalanced response may lead us to novel therapeutic strategies to prevent leprosy reactions.

## Figures and Tables

**Table 1:** Clinical and epidemiological variables of reactional and reaction-free leprosy patients.

		I/TT				BT (PB)				BT(MB)				BB				BL				LL				Total of groups				Total	
		Leprosy reaction		Reaction-free		Leprosy reaction		Reaction-free		Leprosy reaction		Reaction-free		Leprosy reaction		Reaction-free		Leprosy reaction		Reaction-free		Leprosy reaction		Reaction-fee		Leprosy reaction		Reaction-free			
		*n*	*%*	*n* *°*	*%*	*n*	*%*	*n*	*%*	*n*	*%*	*n*	*%*	*n*	*%*	*n*	*%*	*n*	*%*	*n*	*%*	*n*	*%*	*n*	*%*	*n*	*%*	*n*	*%*	*n*	*%*
Operational classification	PB	0	*0*	6	*100*	0	*0*	2	*100*	0	*0*	0	*0*	0	*0*	0	*0*	0	*0*	0	*0*	0	*0*	0	*0*	**0**	*0*	**8**	*47*	*8*	*23.6*
	MB	0	*0*	0	*0*	0	*0*	0	*0*	1	*20*	4	*80*	4	*80*	1	*20*	3	*60*	2	*40*	9	*81.8*	2	*18*	**17**	*100*	**9**	*53*	*23*	*67.6*
Leprosy reaction	T1R									1	*5.9*			4	*23.5*			2	*11.8*			0	*0*			**7**	*41.2*			7	20.5
	ENL									0	*0*			0	*0*			1	*5.9*			9	*53*			**10**	*58.8*			10	29.4
Period of occurrence of leprosy reaction	Before treatment									1	*5.9*			3	*17.6*			1	*5.9*			0	*0*			**5**	*29.4*			5	14.7
	During treatment									0	*0*			0	*0*			0	*0*			0	*0*			**0**	*0*			0	0
	After treatment									0	*0*			1	*5.9*			2	*11.8*			9	*53*			**12**	*70.6*			12	35.2
Disability grade	0	0	*0*	6	*100*	0	*0*	2	*100*	1	*20*	3	*60*	1	*20*	0	*0*	1	*20*	2	*40*	5	*46*	0	*0*	**8**	23.5	**13**	38.2	21	61.7
	1	0	*0*	0	*0*	0	*0*	0	*0*	0	*0*	1	*20*	1	*20*	1	*20*	1	*20*	0	*0*	3	*27*	2	*18*	**5**	14.7	**4**	11.8	9	26.4
	2	0	*0*	0	*0*	0	*0*	0	*0*	0	*0*	0	*0*	2	*40*	0	*0*	1	*20*	0	*0*	1	*9*	0	*0*	**4**	11.8	**0**	0	4	11.7
Sex	Male	0	*0*	0	*0*	0	*0*	2	*100*	1	*20*	4	*80*	2	*40*	1	*20*	2	*40*	2	*40*	5	*45.5*	1	*9.1*	**10**	58.8	**10**	58.8	20	58.8
	Female	0	*0*	6	*100*	0	*0*	0	*0*	0	*0*	0	*0*	2	*40*	0	*0*	1	*20*	0	*0*	4	*36.3*	1	*9.1*	**7**	41.2	**7**	41.2	14	41.2
Age group	18-24	0	*0*	0	*0*	0	*0*	0	*0*	0	*0*	0	*0*	1	*20*	0	*0*	0	*0*	0	*0*	0	*0*	0	*0*	**1**	2.9	**0**	0.0	1	2.9
	25-34	0	*0*	2	*33*	0	*0*	0	*0*	0	*0*	1	*20*	1	*20*	0	*0*	0	*0*	0	*0*	1	*9.09*	0	*0*	**2**	5.9	**3**	8.8	5	14.7
	35-44	0	*0*	3	*50*	0	*0*	0	*0*	0	*0*	0	*0*	1	*20*	1	*20*	2	*40*	0	*0*	2	*19*	1	*9*	**5**	14.7	**5**	14.7	10	29.4
	45-54	0	*0*	0	*0*	0	*0*	1	*50*	0	*0*	1	*20*	1	*20*	0	*0*	0	*0*	1	*20*	3	*27.3*	1	*9*	**4**	11.8	**4**	11.8	8	23.6
	55-64	0	*0*	1	*17*	0	*0*	0	*0*	0	*0*	0	*0*	0	*0*	0	*0*	0	*0*	0	*0*	3	*27.3*	0	*0*	**3**	8.8	**1**	2.9	4	11.7
	≥65	0	*0*	0	*0*	0	*0*	1	*50*	1	*20*	2	*40*	0	*0*	0	*0*	1	*20*	1	*20*	0	*0*	0	*0*	**2**	5.9	**4**	11.8	6	17.7
	Total	**0**	**0**	**6**	**35.2**	**0**	**0**	**2**	**11.8**	**1**	**5.9**	**4**	**23.5**	**4**	**23.5**	**1**	**5.9**	**3**	**17.6**	**2**	**11.8**	**9**	**53**	**2**	**11.8**	***17***	*50*	***17***	*50*	*34*	*100*

I: indeterminate leprosy; TT: tuberculoid leprosy; BT: borderline tuberculoid; BB: borderline borderline; BL: borderline lepromatous; LL: lepromatous leprosy; PB: paucibacillary; MB: multibacillary;

**Table 2:** Correlation matrix among dependent and independent variables of the leprosy reaction cases, based on Pearson's correlation.

Variables	Leprosy reaction					
	IL-10			Toll-like receptor 2		
	*r*_*xy*_	CI (95%)	*p* value	*r*_*xy*_	CI (95%)	*p* value
^∗^Number of injured nerves	0.62	0.13–0.87	0.018	0.47	-0.08–0.80	0.087
^∗^Number of skin lesions	0.69	0.26–0.90	0.005	0.69	0.26–0.90	0.005
^∗^DG	0.28	0.29–0.71	0.331	0.13	-0.42–0.62	0.639
Age	-0.36	-0.75–0.20	0.193	-0.30	-0.72–0.27	0.282
Toll-like receptor 1	0.43	-0.13–0.78	0.121	0.44	-0.11–0.79	0.109
Toll-like receptor 2	0.89	0.68–0.96	<0.001			
IL-10				0.89	0.68–0.96	<0.001
IFN-*γ*	0.05	-0.54–0.61	0.877	0.37	0.52–0.94	0.233
IL-4	-0.17	-0.64–0.40	0.561	0.09	-0.46–0.60	0.747
TNF-*α*	-0.031	-0.55–0.51	0.916	-0.15	-0.63–0.41	0.605
Anti-LAM	0.55	0.03–0.84	0.043	0.70	0.29–0.90	0.004
Anti-PGL-I	0.70	0.08–0.85	0.004	0.68	0.14–0.87	0.007
Bacterial index	0.21	-0.36–0.67	0.468	0.20	-0.36–0.66	0.478

*r*_*xy*_: Pearson's correlation coefficient; CI (95%): confidence interval of 95%, DG: disability grade; IL-10: interleukin 10; IFN-*γ*: interferon gamma; IL-4: interleukin 4; TNF-*α*: tumor necrosis factor (alpha); anti-LAM: anti-lipoarabinomannan antibody; anti-PGL-I: anti-phenolic glycolipid-I antibody. ^∗^Diagnostic data.

**Table 3:** Correlation matrix among dependent and independent variables of the reaction-free leprosy patients group, based on Pearson's correlation.

Variables	Reaction-free group								
	IFN-*γ*			Toll-like receptor 1			Toll-like receptor 2		
	*r*_*xy*_	CI (95%)	*p* value	*r*_*xy*_	CI (95%)	*p* value	*r*_*xy*_	CI (95%)	*p* value
^∗^Number of injured nerves	0.04	-0.68–0.72	0.905	-0.20	-0.77–0.62	0.576	-0.18	-0.63–0.76	0.606
^∗^Number of skin lesions	-0.35	-0.85–0.47	0.265	-0.26	-0.74–0.67	0.473	-0.47	-0.86–0.43	0.142
DG	-0.06	-0.78–0.61	0.869	-0.09	-0.82–0.54	0.795	-0.11	-0.71–0.70	0.741
Age	0.24	-0.55–0.81	0.513	0.07	-0.64–0.76	0.845	-0.06	-0.62–0.77	0.865
Toll-like receptor 1	0.73	0.03–0.94	0.023				0.87	0.43–0.98	0.002
Toll-like receptor 2	0.78	0.45–0.98	0.013	0.87	0.43–0.98	0.002			
IL-10	0.66	-0.13–0.95	0.053	0.32	-0.62–0.84	0.401	0.43	-0.55–0.87	0.249
IFN-*γ*				0.74	0.03–0.94	0.023	0.78	0.45–0.98	0.013
IL-4	-0.31	-0.85–0.47	0.384	-0.25	-0.84–0.49	0.485	-0.34	-0.86–0.44	0.309
TNF-*α*	-0.28	-0.85–0.45	0.426	-0.31	-0.87–0.38	0.386	-0.36	-0.87–0.41	0.279
Anti-LAM	-0.29	-0.84–0.49	0.419	-0.28	-0.85–0.47	0.429	-0.28	-0.82–0.54	0.399
Anti-PGL-I	-0.51	-0.89–0.31	0.132	-0.33	-0.78–0.62	0.357	-0.35	-0.86–0.42	0.294
Bacterial index	-0.45	-0.87–0.38	0.197	-0.38	-0.81–0.55	0.283	-0.74	-0.89–0.33	0.144

*r*_*xy*_: Pearson's correlation coefficient; CI (95%): confidence interval of 95%, DG: disability grade; IL-10: interleukin 10; IFN-*γ*: interferon gamma; IL-4: interleukin 4; TNF-*α*: tumor necrosis factor (alpha); anti-LAM: anti-lipoarabinomannan antibody; anti-PGL-I: anti-phenolic glycolipid-1 antibody. ^∗^Diagnostic data.

## Data Availability

The path analysis and laboratory data of all patients used to support the findings of this study are available from the corresponding author upon request.
